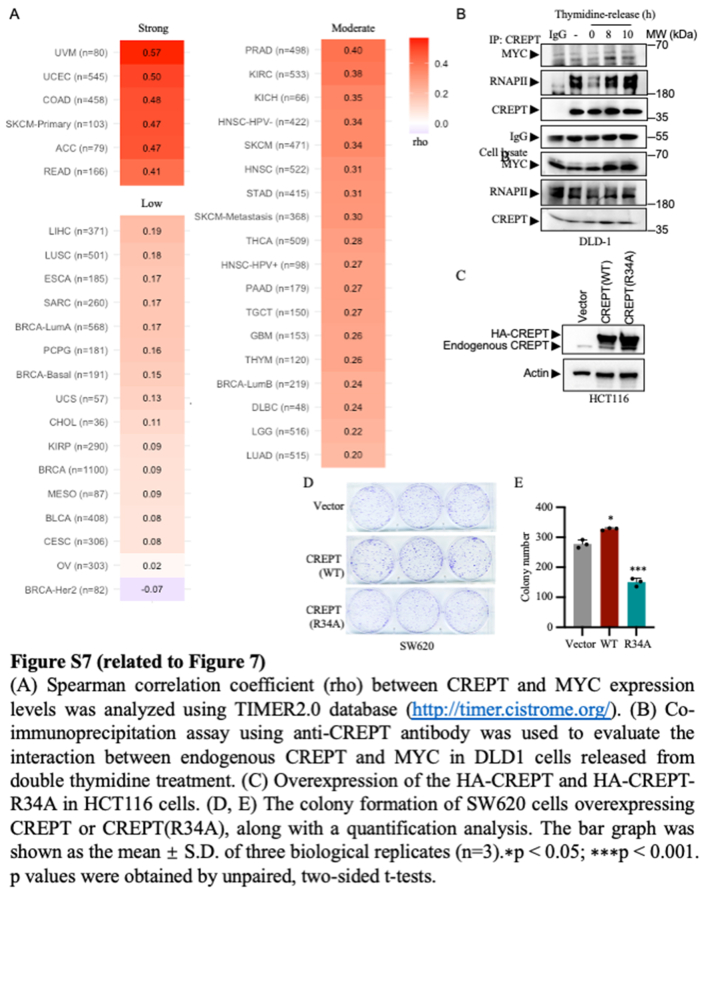# An oncoprotein CREPT functions as a co-factor in MYC-driven transformation and tumor growth

**DOI:** 10.1016/j.jbc.2024.108030

**Published:** 2024-11-29

**Authors:** Mengdi Li, Jingya Li, Chunhua He, Guancheng Jiang, Danhui Ma, Haipeng Guan, Yuting Lin, Meng Li, Jing Jia, Xiaolin Duan, Yinyin Wang, Fangli Ren, Haitao Li, Xiaoguang Wang, Chenxi Cao, Zhijie Chang

**Affiliations:** 1 State Key Laboratory of Membrane Biology, School of Medicine, Tsinghua University, Beijing, China; 2 Department of Surgery, The Second Affiliated Hospital of Jiaxing University, Jiaxing, China; 3 MOE Key Laboratory of Protein Sciences, Beijing Frontier Research Center for Biological Structure, School of Medicine, Tsinghua University, Beijing, China; 4 Department of Medicine, Zhuhai Hospital of Integrated Traditional Chinese and Western Medicine, Zhuhai, China

**Keywords:** MYC, CREPT, RNA polymerase II (RNAPII), transcription, colon cancer

## Abstract

Understanding the mechanisms behind MYC-driven oncogenic transformation could pave the way for identifying novel drug targets. This study explored the role of CREPT in MYC-induced malignancy by generating MYC-transformed mouse embryonic fibroblasts (MEFs) with conditional CREPT deletion. Our results demonstrated that the loss of CREPT significantly impaired MYC-induced colony formation and cell proliferation, indicating that CREPT is essential for the malignant transformation of MEFs. Reintroducing CREPT in CREPT-deficient cells restored malignant properties. Furthermore, CREPT overexpression alone enhanced colony formation upon MYC induction but was insufficient to induce transformation without MYC, suggesting a cooperative interaction between CREPT and MYC in malignant transformation. CREPT deletion resulted in delayed cell cycle progression during the G2/M and S phases. CREPT enhanced the expression of MYC target genes by directly interacting with MYC through the CID domain of CREPT and the PEST domain of MYC. Arginine 34 of CREPT was identified as a critical residue for the interaction with MYC, and its mutation lost the ability of CREPT to promote MYC-driven colony formation and tumor growth in colorectal cancer models. Additionally, CREPT facilitated the recruitment of RNA Polymerase II to MYC-binding promoters, promoting transcriptional initiation of MYC-targeted genes. Our study also revealed a strong correlation between CREPT and MYC expression in various human cancers, particularly in colorectal cancer, where their interaction appears to play a significant role in tumorigenesis. These findings suggest that the CREPT-MYC interaction is crucial for the progression of MYC-driven cancers and presents a potential target for therapeutic intervention.

The MYC proto-oncogenes, including *MYC*, *MYCN*, and *MYCL*, belong to the superfamily of basic helix-loop-helix-leucine zipper (bHLHZip) proteins (1). *MYC* is dysregulated in at least 70% of human cancers and is considered a primary driver of various cancers, particularly colorectal carcinomas (2, 3). MYC expression is frequently associated with a poor prognosis and severe cancer aggression. To date, no MYC-targeting inhibitors have been approved for clinical use. While numerous studies suggest that inhibiting MYC could offer a clinically significant therapeutic opportunity, various approaches are still under investigation to target MYC directly or indirectly (4).

Compared to MYCN and MYCL, which are expressed in a tissue-restricted manner, MYC is ubiquitously expressed and typically elevated in proliferating cells (5). MYC proteins serve as universal amplifiers of transcription, facilitating the running of cellular processes. As transcription factors, MYC proteins form heterodimers with MAX to bind a palindromic DNA motif (CACGTG), known as ‘E-box’, for the regulation of gene transcription to orchestrate a wide array of cellular functions including cell growth, proliferation, differentiation, and metabolism (6, 7). MYC interacts with over 300 partners, including histone acetyltransferase (HAT) complexes and more than 100 transcription factors such as general transcription factor IIF (TFIIF) (7, 8, 9). These interactions render chromatin accessible for RNA polymerase II (RNAPII) loading and recruitment, thereby facilitating transcription initiation and the release of RNAPII from its paused state. However, the exact mechanisms that govern the role and dynamics of MYC in initiating cancer cells remain unclear. Despite the prevailing view that most molecular interactions with MYC result from unproductive encounters, it is possible that a specific factor may retain MYC into a particular complex to effectively regulate gene transcription in tumor cells.

CREPT (also named RPRD1B) is abundantly expressed in multiple human cancers, including colorectal, gastric, and non-small lung cancers, with poor prognosis and short survival time (10, 11, 12). Our initial study demonstrated that CREPT promoted tumor cell proliferation by facilitating the transcription of tumor-related genes (13). We previously showed that CREPT enhanced Wnt signaling activation by binding to β-catenin and TCF4, to promote cell proliferation and invasion (14). Recently, we observed that CREPT coordinated with p300 to modulate histone modification and promoted gene accessibility for the RNAPII complex (15). CREPT contains a C-terminal domain (CTD)-interacting domain (CID), which mediates direct interaction with RNAPII and is often present in proteins regulating gene transcription and RNA processing (16, 17). Previous studies showed that CREPT promoted the RNAPII occupancy on the *CCND1* gene and possessed the ability to modulate the phosphorylation and acetylation of CTD on RNAPII by cooperating with phosphatase RNA polymerase II-associated protein 2 (RPAP2) and histone deacetylase 1 (HDAC1) (10, 17, 18). Therefore, it is likely that CREPT acts as a transcriptional co-factor that regulates the activity of RNAPII in genes related to tumor malignancy.

Given the significant role of CREPT in transcription regulation and its elevated expression in tumor cells, we hypothesized that CREPT might be involved in the transcriptional activity of MYC in cancer cells. In this study, we identify a direct interaction between CREPT and MYC and their collaborative role in gene transcription regulation. Our results indicate that CREPT is crucial for the recruitment of RNAPII to MYC-responsive promoters, facilitating the initiation of gene transcription. These findings highlight CREPT as a potential novel target for treating tumors with MYC overexpression.

## Results

### CREPT is required for the malignancy of MYC-transformed MEFs

**Figure 1:**
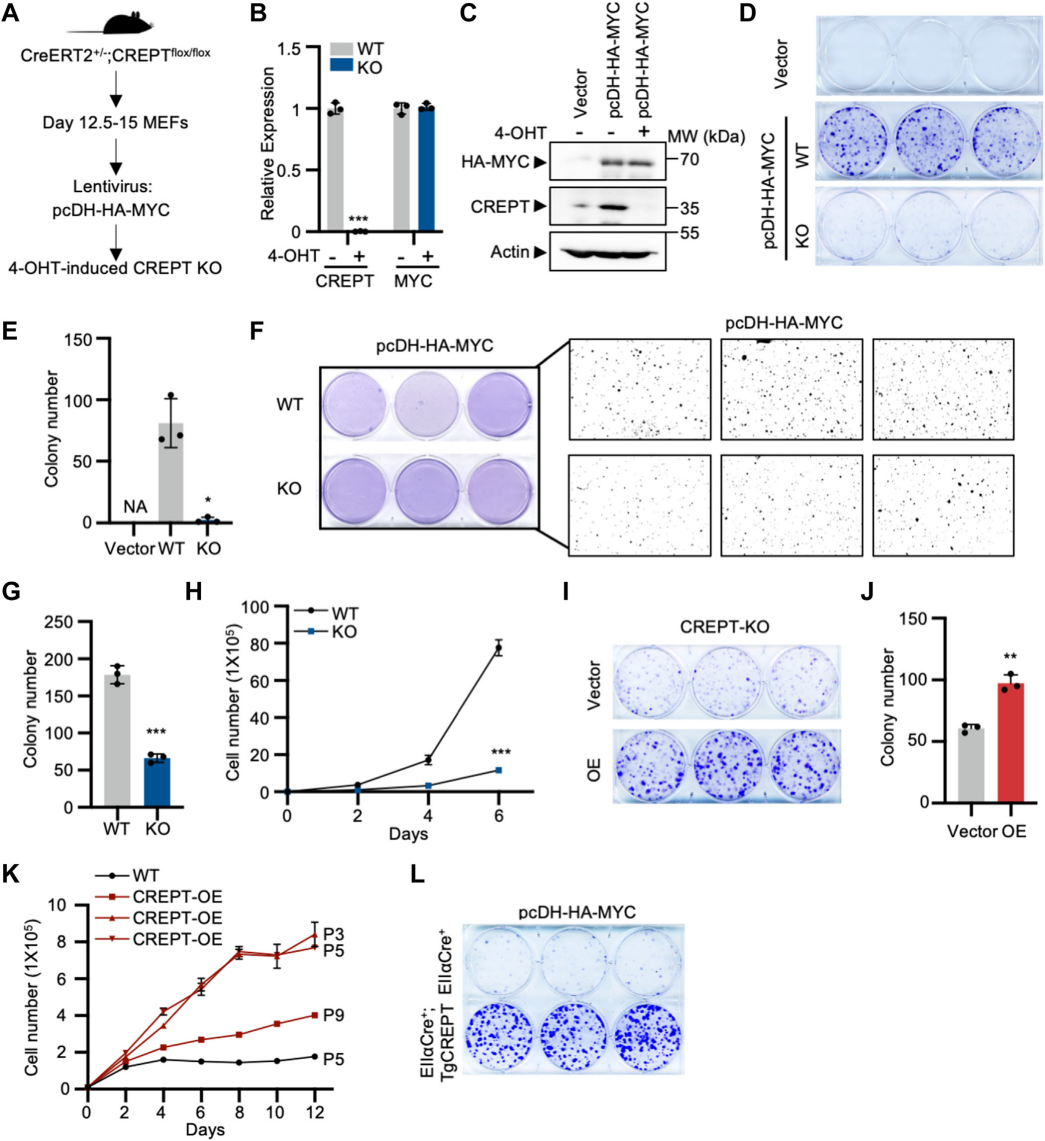
**CREPT is critical for the colony formation and proliferation of MYC-transformed MEFs.***A*, a schematic diagram illustrating the generation of CreERT2^+/−^;CREPT^flox/flox^ mouse embryo fibroblasts (MEFs) and their transformation with lentivirus carrying pcDH/pcDH-HA-MYC. MEFs were isolated from day 12.5 to 15 mouse embryos and subsequently infected with the lentivirus. CREPT knockout (KO) was induced using 1 μM 4-Hydroxytamoxifen (4-OHT). *B* and *C*, the mRNA and protein levels of CREPT and exogenous MYC in CREPT wild-type (WT) and knockout (KO) MYC-transformed MEFs were tested using RT-qPCR and Western blotting. 4-OHT successfully induced CREPT-deficiency in MEFs without affecting the expression of exogenous MYC. *D* and *E*, representative images of colony formation of CREPT WT and KO MYC-transformed MEFs, along with the corresponding quantification results. *F* and *G*, representative images of soft agar colony formation of CREPT WT and KO MYC-transformed MEFs, along with the corresponding quantification results. *H*, cell growth is evaluated by counting cell number. *I* and *J*, overexpression of exogenous GFP-CREPT in CREPT-deficient MYC-transformed MEFs, along with the corresponding quantification results. *K*, cell growth of different passages of MEFs obtained from EIIαCre^+^ or EIIαCre^+^;Tg-CREPT (CREPT-over-expressed mouse) mice were tested. *L*, MEFs obtained from EIIαCre^+^ or EIIαCre^+^;Tg-CREPT mice were transformed by lentivirus carrying pcDH-HA-MYC. CREPT over-expressed cells showed markedly increased colony formation than the control cells. Each column of the bar graph represents the mean ± S.D. of three biological replicates (n = 3). ∗*p* < 0.05; ∗∗*p* < 0.01; ∗∗∗*p* < 0.001. *p* values were obtained by unpaired, two-sided t-tests.

**Fig. S1:**
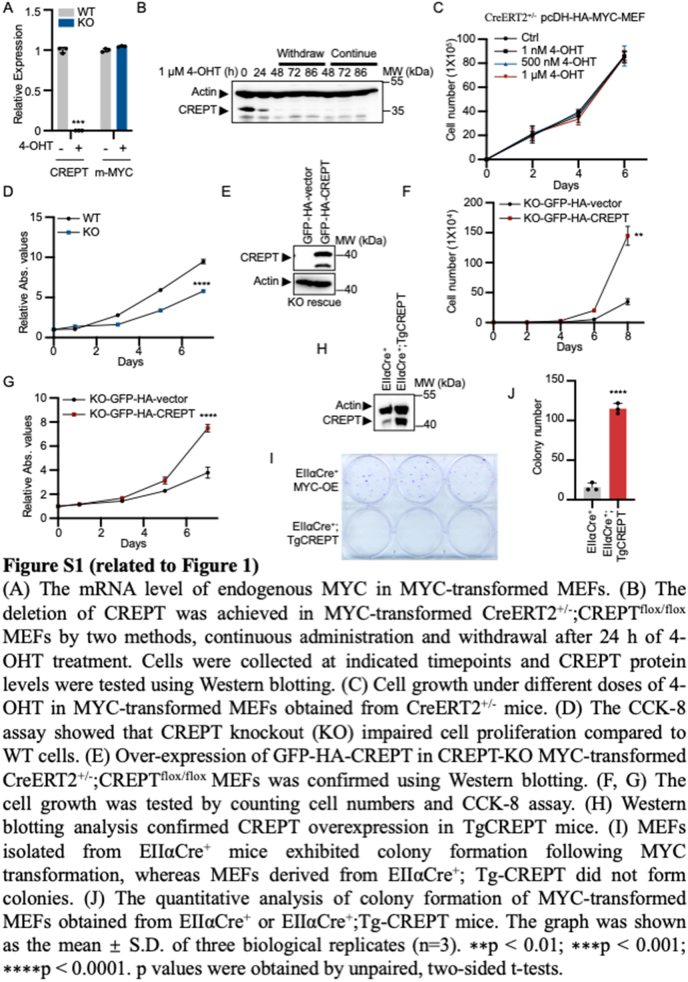


To investigate the role of CREPT in MYC-regulated cellular activities, we established a stable cell line based on MYC expression in primary embryonic fibroblasts (MEFs) obtained from CreERT2^+/−^;CREPT ^flox/flox^ mice (Fig. 1*A*). CREPT was deleted by the addition of 4-OHT in this cell line, while both the exogenous and endogenous MYC expression were not altered, as assayed by RT-qPCR and Western blotting analyses (Figs. 1, *B* and *C*; S1, *A* and *B*). Notably, different concentrations of 4-OHT (1, 500, and 1000 nM) did not affect the proliferation of MYC-transformed MEFs from CreERT2^+/−^;CREPT^WT/WT^ mice, where CREPT was not deleted (Fig. S1*C*), indicating that 4-OHT *per se* had no discernible effect on cell growth. Intriguingly, a colony formation assay showed that the addition of 4-OHT for 24 h caused the failure of colony formation in MYC-transformed MEFs (Fig. 1, *D* and *E*). This result suggests that deletion of CREPT impaired the MYC-induced transformation of MEFs, which was further confirmed by a soft agar assay (Fig. 1, *F* and *G*). Consistently, a cell proliferation experiment, measured by both cell numbers (Fig. 1*H*) and the CCK-8 assay (Fig. S1*D*), showed that the deletion of CREPT slowed down cell proliferation. Collectively, these results suggest that CREPT is essential for the ability to form colonies and enhance cell proliferation during MYC-induced MEF transformation. To validate the impact of CREPT deletion, we re-introduced CREPT expression in MYC-transformed MEFs in which CREPT was deleted by the addition of 4-OHT (CREPT-KO) (Fig. S1*E*). The results demonstrated that both colony formation and cell proliferation rates were restored in the CREPT-rescued cells compared to the CREPT-deficient cells (Figs. 1, *I* and *J*; S1, *F* and *G*). These results suggest that CREPT is critical for the malignant features of MYC-transformed MEFs.

To address whether CREPT can mediate cell transformation independently of MYC, as it was reported to induce NIH 3T3 cells to form tumors (10), we isolated MEFs from EIIαCre^+^;Tg-CREPT mice that over-express CREPT (Fig. S1*H*). A cell proliferation assay showed that wild-type (WT) MEFs terminated growth (appeared to undergo senescence) after the fifth passage (P5), consistent with other reports (19); however, MEFs from EIIαCre^+^;Tg-CREPT mice displayed robust proliferation capabilities beyond the fifth passage (Fig. 1*K*), implying that over-expression of CREPT delays cellular senescence. In contrast, we observed that MEFs from EIIαCre^+^;Tg-CREPT mice failed to form colonies (Fig. S1*I*), indicating that CREPT alone is insufficient to induce MEF transformation in the absence of MYC. However, MEFs from EIIαCre^+^;Tg-CREPT mice formed significantly more colonies upon MYC induction (Figs. 1*L* and S1*J*). These results indicate that both CREPT and MYC are essential for MEF transformation, implying their cooperative role in the malignant alteration of normal cells.

### Loss of CREPT prolongs cell cycle progress in MYC-transformed MEFs

**Figure 2:**
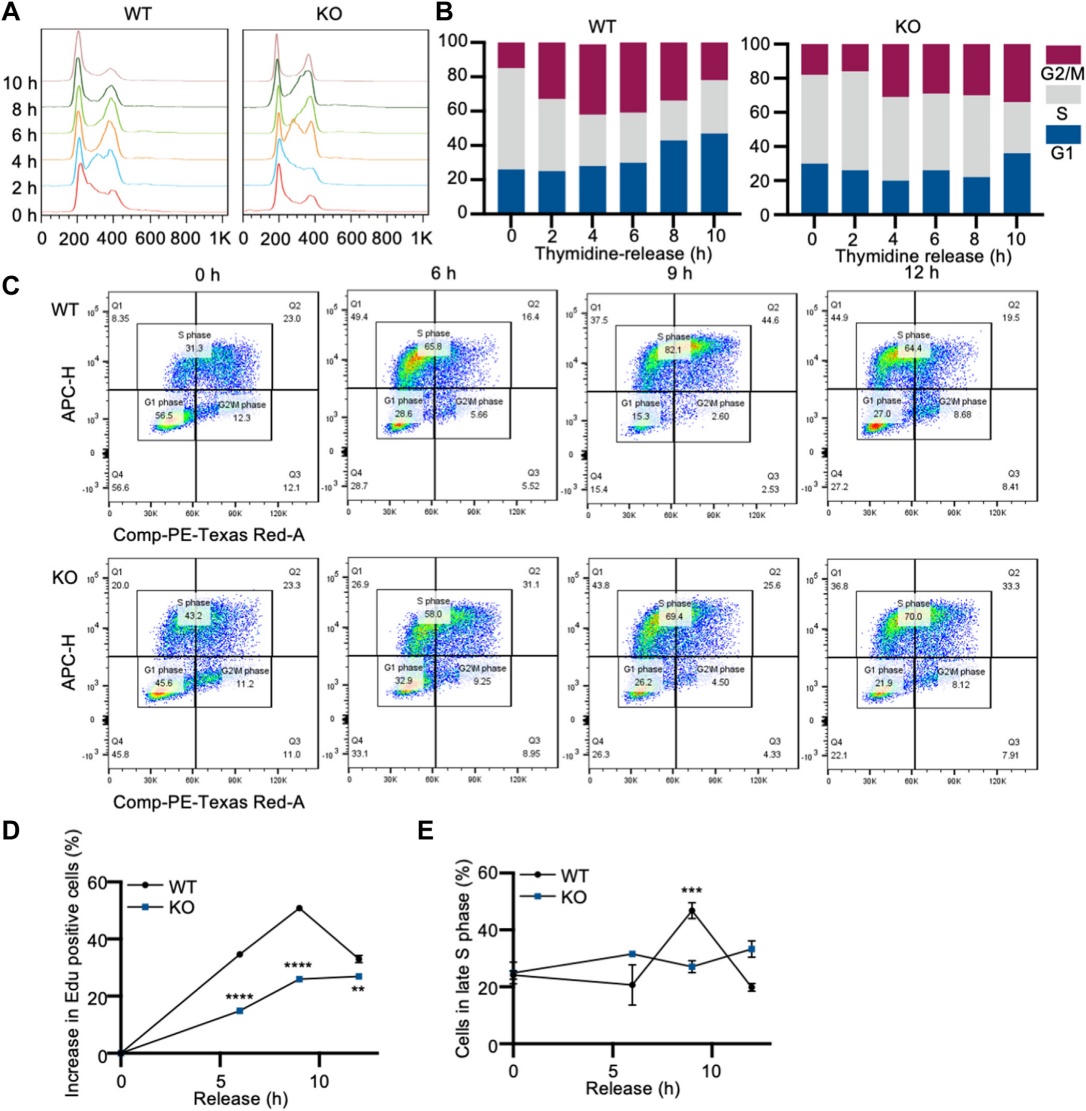
**Loss of CREPT prolongs cell cycle in MYC-transformed MEFs.***A* and *B*, flow cytometry analyses of cell cycle using propidium iodide (PI) staining after releasing from double thymidine block (DTB). *C*, flow cytometry analysis was performed to evaluate the cell cycle of cells released from starvation with 0.5% FBS. Cells were incubated with EdU and stained with PI and anti-EdU antibody. *D*, a quantification analysis of increase in EdU positive cells. *E*, a quantification analysis of cell proportion in the late S phase. The line graph was shown as the mean ± S.D. of three biological replicates (n = 3). ∗∗*p* < 0.01; ∗∗∗*p* < 0.001; ∗∗∗∗*p* < 0.0001. *p* values were obtained by unpaired, two-sided t-tests.

**Fig. S2:**
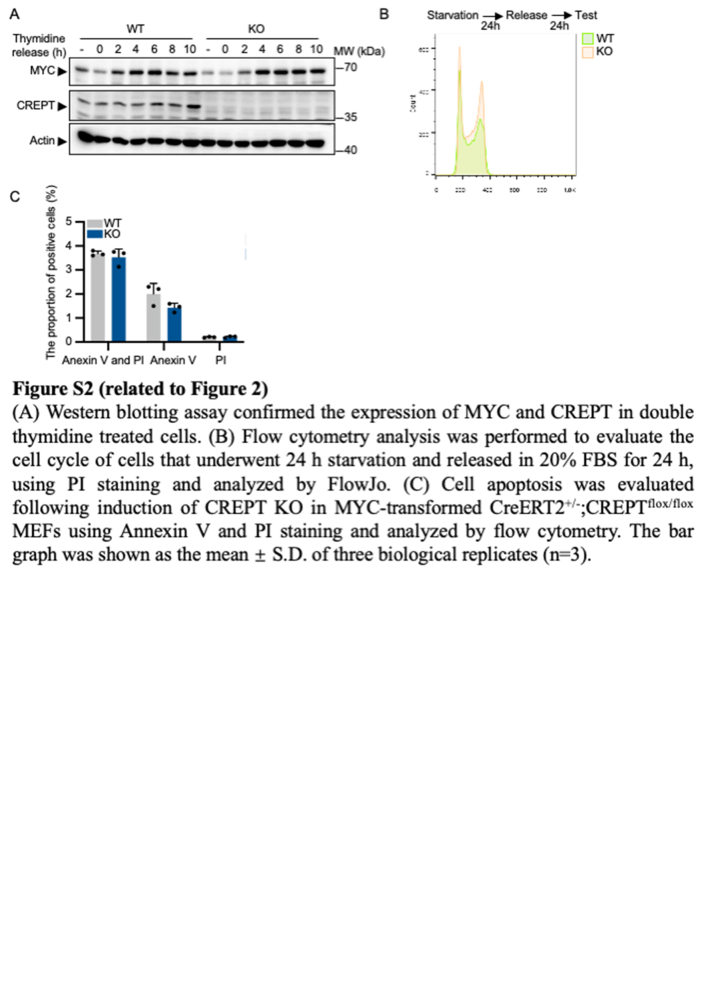


To determine how CREPT coordinates MYC during MEF transformation, we determined whether the cell cycle was altered. To this end, we synchronized the cells in the G1/S phase with 2 mM thymidine. Western blotting analysis showed that CREPT was efficiently deleted, but the CMV-driven MYC expression was not affected in MYC-transformed MEFs (Fig. S2*A*). FACS analysis showed that wild-type cells entered the G2/M phase 2 h after thymidine release, with approximately 40% of cells in the G2/M phase at 6 h (Fig. 2, *A* and *B*, left panel). In contrast, CREPT-deficient (KO) cells entered the G2/M phase at 4 h and remained at this stage until 10 h (Fig. 2, *A* and *B*, right panel). Notably, the WT cells re-entered the G1 phase at 8 h, but the CREPT-deficient cells were retarded at 10 h, with a delay period of 2 h (Fig. 2, *A* and *B*). In a separate experiment using a starvation synchronization strategy, we observed that CREPT-deficient cells were arrested in the G2/M phase (Fig. S2*B*). Overall, these results indicate that deletion of CREPT delays cell cycle progression by 2 h during the G2/M and G1 phases. These results are consistent with those of our previous study (20).

To investigate whether CREPT regulates S phase progression, we synchronized MYC-transformed MEFs under starvation conditions and subsequently released the cells with 20% FBS to induce their entry into the S phase. FACS analysis showed that at 0 h, the majority of cells were in the G1 phase (56.5%), and after 6 h, the cells entered the early S phase (49.4%), followed by progression into the late S phase at 9 h (44.6%) (Fig. 2*C*, WT). However, under conditions of CREPT deficiency, 43.8% of the cells remained in the early S phase at 9 h (Fig. 2*C*, KO). This finding indicates that deletion of CREPT delayed cell cycle progression, with a 3 h difference, as noted in the appearance of the early S phase peaks (6 h with 49.4% vs. 9 h with 43.8% in Fig. 2*C*). On the other hand, we observed that more cells were distributed in the early S phase with fewer G0/G1 phase cells in the CREPT deficiency cell than wild-type cells after starvation (Fig. 2*C* and 0 h), suggesting that CREPT may also affect the arrest of cells at G0/G1 phase. Overall, we calculated the percentage of cells in the S phase. The results showed that while WT cells exhibited a peak in the late S phase 9 h after starvation release, CREPT-deficient cells failed to display any noticeable peak (Fig. 2*D*). Similarly, the percentage of cells in the late S phase peaked at 9 h in WT cells but remained relatively unchanged in CREPT-deficient cells (Fig. 2*E*). In contrast, we observed that the deletion of CREPT had no substantial impact on apoptosis in MYC-transformed MEFs (Fig. S2*C*). These findings collectively indicate that CREPT functions not only in the G2/M phase but also in the S phase process in MYC-transformed cells.

### Deletion of CREPT represses the MYC target gene transcription

**Figure 3:**
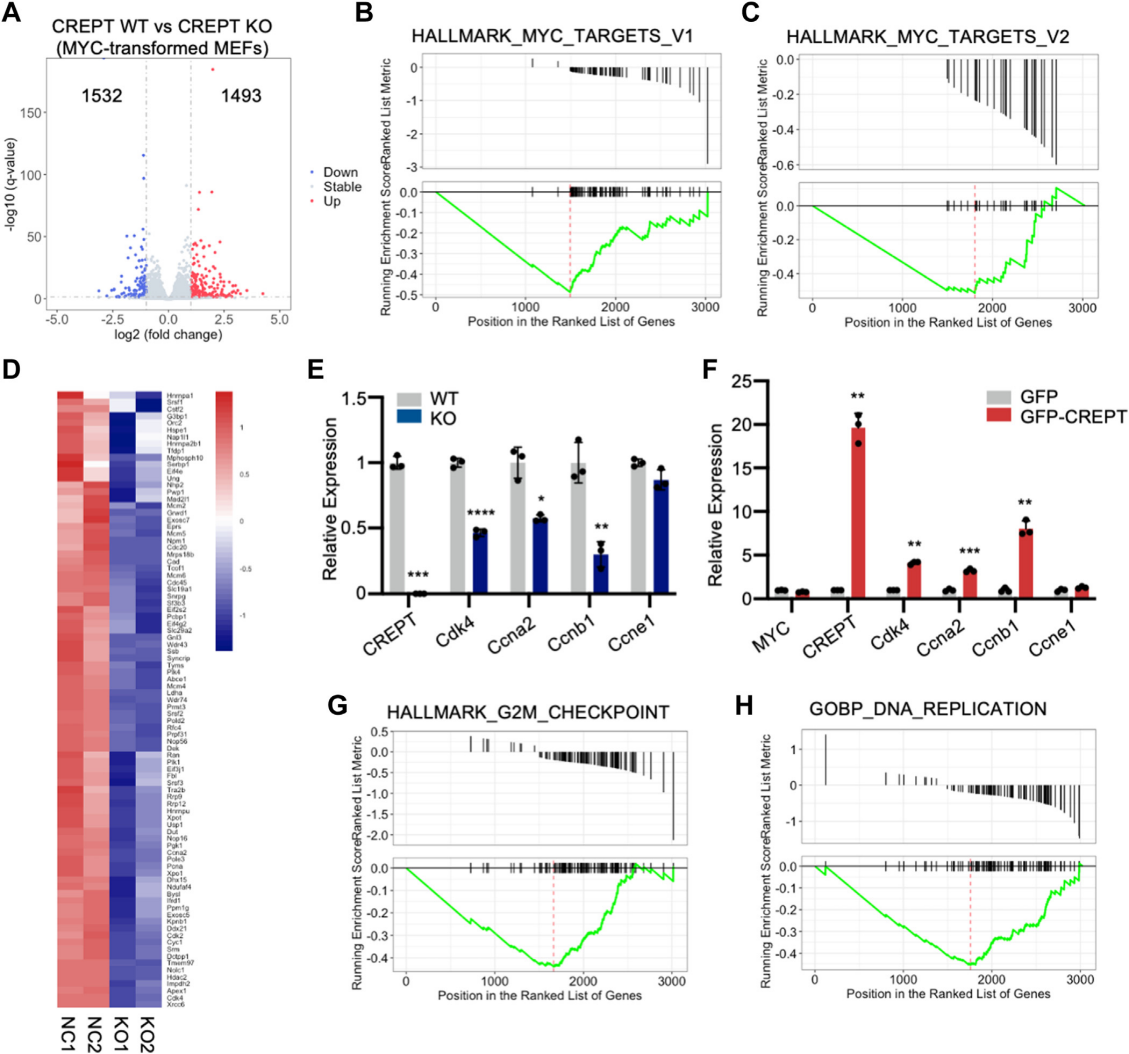
**Deletion of CREPT represses MYC target gene transcription.***A*, a volcano plot displays the differential expression of genes (DEGs) in CREPT-deleted transformed MEFs compared to the control group. The plot represents 3025 genes whose expression was altered, with 1532 downregulated genes and 1493 upregulated genes (padj<0.05). *B* and *C*, Gene Set Enrichment Analysis (GSEA) for DEGs from CRETP WT and KO MYC-transformed MEFs showed that the DEGs were negatively associated with MYC target gene sets. *D*, a heatmap of MYC target genes in CRETP WT and KO MYC-transformed MEFs. *E*, the mRNA levels of CDK4, *Ccna2*, *Ccnb1* and *Ccne1* in CRETP WT and KO transformed MEFs were confirmed by RT-qPCR. *F*, RT-qPCR was employed to assess the mRNA levels of CREPT, CDK4, *Ccna2*, *Ccnb1*, *Ccne1* and exogenous MYC in CRETP KO and CREPT-rescued MYC-transformed MEFs. *G* and *H*, GSEA analysis for DEGs showing negative correlations with G2/M checkpoint and DNA replication. Each column of the bar graph represents the mean ± S.D. of three biological replicates (n = 3). ∗*p* < 0.05; ∗∗*p* < 0.01; ∗∗∗*p* < 0.001; ∗∗∗∗*p* < 0.001. *p* values were obtained by unpaired, two-sided t-tests.

To elucidate the cooperative mechanism underlying the regulation of cell cycle progression by CREPT and MYC, we performed RNA-seq analysis to profile gene expression alterations in MYC-transformed MEFs. A comparative analysis revealed significant alterations in gene expression in MYC-transformed MEFs under WT and CREPT-deficient conditions (Fig. 3*A*, adjusted *p*-value < 0.05). Among these differentially expressed genes (DEGs), 1532 were downregulated and 1493 were upregulated (Fig. 3*A*, blue and red). Subsequent Gene Set Enrichment Analysis (GSEA) demonstrated two distinct sets of MYC target genes that were inversely correlated with DEGs (Fig. 3, *B* and *C*). These were categorized as HALLMARK_MYC_TARGETS_V1 (Fig. 3*B*, NES = −2.403, *p*-value = 0.004, FDR = 0.031) and HALLMARK_MYC_TARGETS_V2 (Fig. 3*C*, NES = −1.937, *p*-value = 0.003, FDR = 0.031). A heatmap showed that deletion of CREPT markedly suppressed the expression of these MYC downstream target genes in two independently repeated experiments (Fig. 3*D*).

**Fig. S3:**
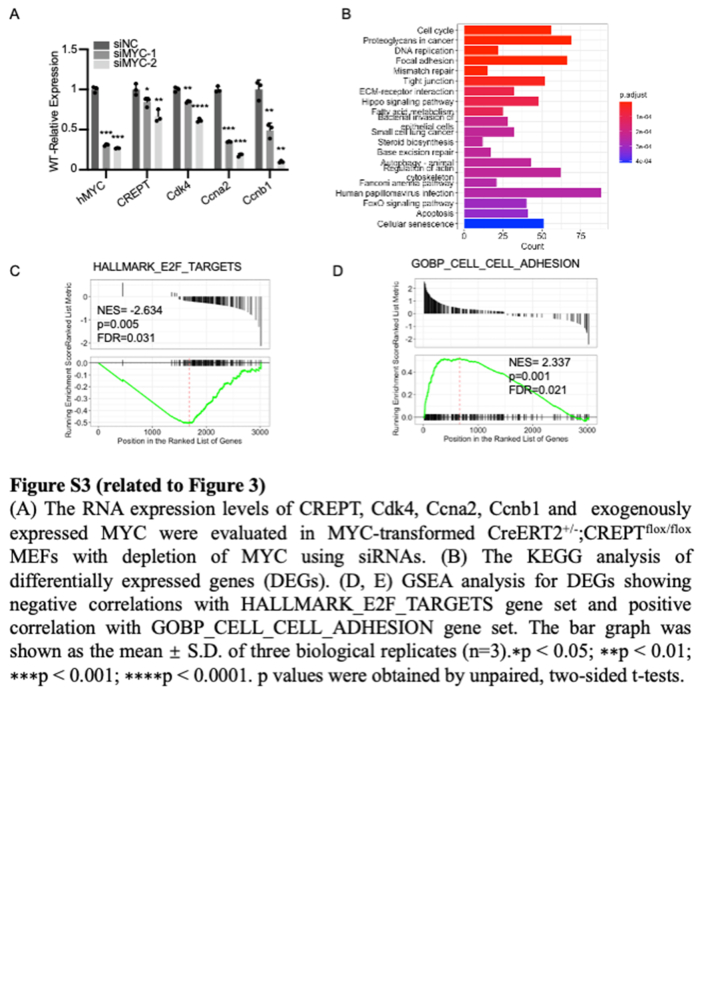


To validate the results of RNA-seq analysis, we selected *Cdk4*, *Ccna2*, and *Ccnb1* genes that were previously identified as downstream targets of MYC (21, 22). RT-qPCR analysis confirmed that deletion of CREPT diminished the expression of these genes, but not of the *Ccne1* gene, which remained unaltered in CREPT-deficient cells, as revealed by our RNA-seq data (Fig. 3*E* and Table S2). Moreover, MYC deficiency induced by the two siRNAs significantly suppressed the expression of *Cdk4*, *Ccna2*, and *Ccnb1* (Fig. S3*A*), indicating that CREPT and MYC contemporaneously modulate these genes. A subsequent rescue assay showed that overexpression of GFP-CREPT markedly restored the expression of *Cdk4*, *Ccna2*, and *Ccnb1*, but not that of *Ccne1* (Fig. 3*F*). Collectively, these findings support the conclusion that CREPT actively enhances the expression of a subset of MYC-regulated genes.

To delineate the impact of CREPT on the regulation of gene expression, we conducted a KEGG pathway analysis. The results showed that the DEGs were predominantly enriched in pathways involved in cell cycle progression, proteoglycan signaling in cancer, DNA replication, focal adhesion, and mismatch repair (Fig. S3*B*). GSEA revealed negative associations between DEGs and gene sets regulating the G2/M checkpoint (Fig. 3*G*), DNA replication (Fig. 3*H*), and E2F activation (Fig. S3*C*), which are the main events in cell cycle progression (Tables S3 and S4). Furthermore, GSEA revealed a significant upregulation of genes associated with cell adhesion in CREPT-deficient cells (Fig. S3*D*), consistent with the KEGG findings (Fig. S3*B*). Collectively, these data indicate that CREPT regulates cell cycle progression and cellular adhesion by modulating the expression of MYC-dependent genes.

### CREPT interacts with MYC *via* CID and PEST domains

**Figure 4:**
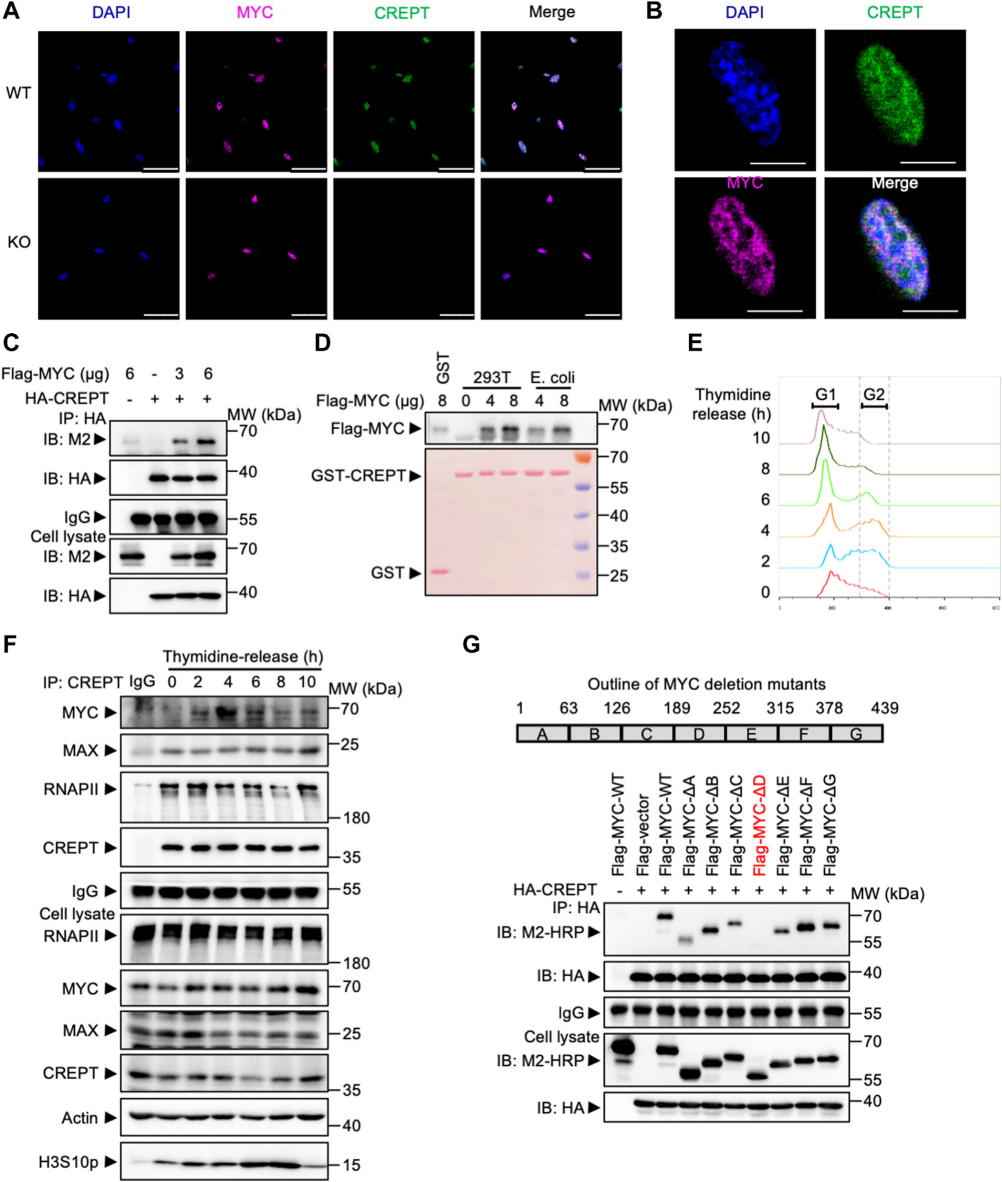
**CREPT interacts with the PEST domain of MYC.***A*, immunofluorescence (IF) staining of CREPT and MYC in transformed MEFs showing deletion of CREPT did not alter the MYC localization using confocal microscopy. *B*, a single nucleus showing the co-localization of CREPT and MYC in a condensed manner using confocal microscopy. *C*, Co-immunoprecipitation with an anti-HA antibody to pull down exogenous expressed HA-CREPT in 293T cells. Western blotting for Flag-MYC showed the amount of Flag-MYC binding to HA-CREPT. *D*, GST-pulldown was employed to evaluate the bindings between GST-CREPT (4 μg) purified from 293T cells or *Escherichia coli* and Flag-MYC (4 or 8 μg) purified from 293T cells. *E*, MYC-transformed MEFs were released from DTB treatment for indicated time points. The cell cycle was determined using PI staining and analyzed by flow cytometry and FlowJo. *F*, the endogenous interaction between CREPT and MYC was assessed by co-immunoprecipitation assay using anti-CREPT antibody in the MYC-transformed MEFs released from DTB. Western blotting for MYC was used to determine the amount of MYC binding to CREPT. *G*, co-immunoprecipitation between HA-CREPT and seven Flag-MYC deletion mutants in CREPT KO 293T cells using an anti-HA antibody. Western blotting showed the amount of Flag-MYC mutants binding to HA-CREPT.

**Fig. S4:**
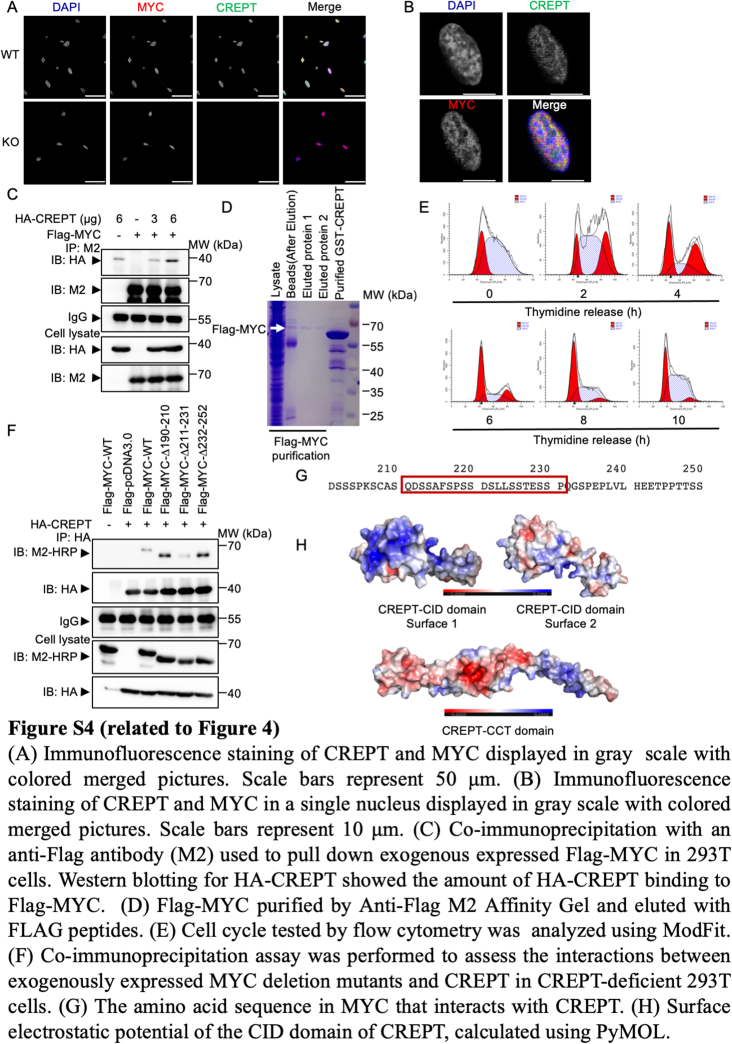


To investigate the regulatory mechanism by which CREPT modulates MYC-dependent gene expression, we examined the physical interaction between CREPT and MYC. Immunofluorescence assays revealed that colocalization of CREPT and MYC occurred in the nucleus, with a near-complete overlap observed in WT MYC-transformed MEFs (Figs. 4*A* and S4*A*). Notably, the deletion of CREPT did not affect the nuclear localization of MYC (Fig. 4*A*, lower panel). An enlarged single nucleus demonstrated that MYC and CREPT colocalized in a condensed manner in the nucleus (Figs. 4*B* and S4*B*). To ascertain whether their nuclear colocalization signified a direct interaction, we conducted co-immunoprecipitation experiments. The results demonstrated that HA-tagged CREPT precipitated Flag-tagged MYC (Fig. 4*C*), and reciprocally, Flag-MYC precipitated HA-CREPT (Fig. S4*C*). To address whether CREPT and MYC directly bind *in vitro*, we performed a GST pull-down assay using GST-tagged CREPT and FLAG-tagged MYC. The results showed that CREPT purified from both mammalian cells and *Escherichia coli* robustly precipitated Flag-tagged MYC (Figs. 4*D* and S4*D*). This finding suggests that CREPT and MYC may have a direct interaction, possibly independent of the phosphorylation state of the CREPT protein. Interestingly, we synchronized the cells by DTB and released the cells at indicated time points, the cells were stained with PI to identify the stage of cells and an endogenous immunoprecipitation assay was performed using the cells at each time point (Figs. 4*E* and S4*E*). The results demonstrated that the CREPT-MYC interaction predominantly occurred during the late S and G2/M phases rather than during the G1 phase (Fig. 4*F*).

To identify the specific region of MYC that interacts with CREPT, we generated MYC deletion mutants. Exogenous immunoprecipitation assay revealed that the absence of region D, which encompasses the PEST domain, notably reduced the binding affinity between CREPT and MYC (Fig. 4*G*, red). Further refinement using three additional MYC truncations identified amino acids 211 to 231 as critical residues for CREPT binding (Fig. S4*F*). These data suggest that the PEST domain, specifically residues 211 to 231, mediates CREPT-MYC interaction. These residues in the PEST domain are composed of serine residues, which can acquire a negative charge through phosphorylation, alongside negatively charged aspartic and glutamic acids (Fig. S4*G*). Concurrently, electrostatic potential mapping *via* PyMOL revealed distinct positive charges on the CID domain and negative charges on the CCT domain of CREPT (Fig. S4*H*). Taken together, these analyses suggest that the electrostatic complementarity between the negatively charged PEST domain region in MYC and the positively charged CREPT surfaces may facilitate their physical interaction.

### Arginine 34 is critical for CREPT binding with MYC

**Figure 5:**
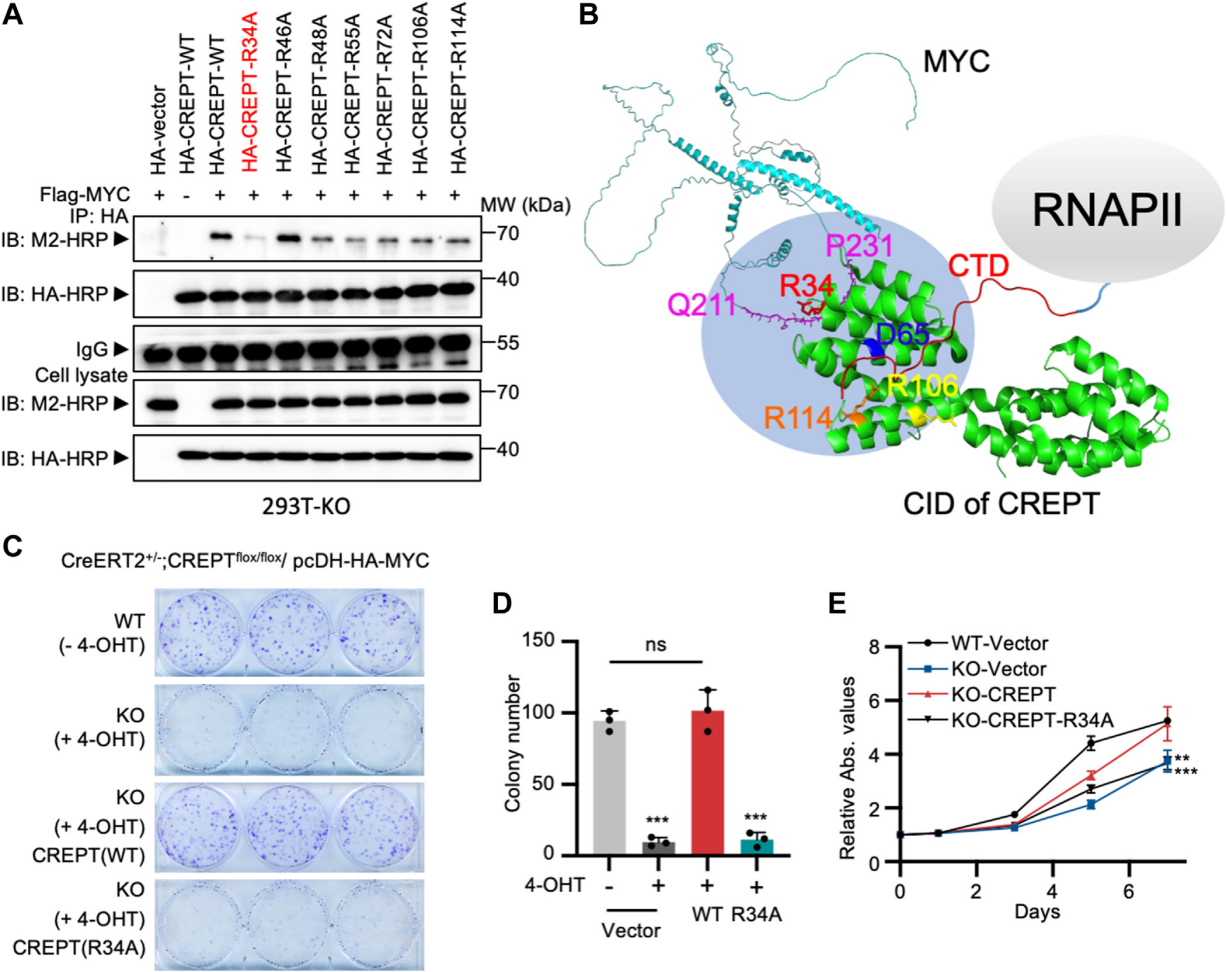
**The importance of arginine 34 for CREPT binding with MYC.***A*, co-immunoprecipitation was performed in CREPT-deficient 293T cells to pull down WT and seven HA-CREPT mutations using an anti-HA antibody. The associated Flag-MYC proteins were examined by a Western blotting using an antibody against Flag. *B*, a protein interaction model of CREPT, MYC and RNAPII. R34 in CREPT interacts with the amino acids from Q211 to P231 in MYC. D65, R106 and R114 in CREPT mediates the binding with CTD of RNAPII. *C* and *D*, the colony formation of CREPT-deficient MYC-transformed MEFs rescued by the over-expression of CREPT but not CREPT(R34A), as confirmed by quantitative analyses. *E*, the cell proliferation of CREPT-deficient MYC-transformed MEFs rescued by the over-expression of CREPT but not CRPET(R34A).

**Fig. S5:**
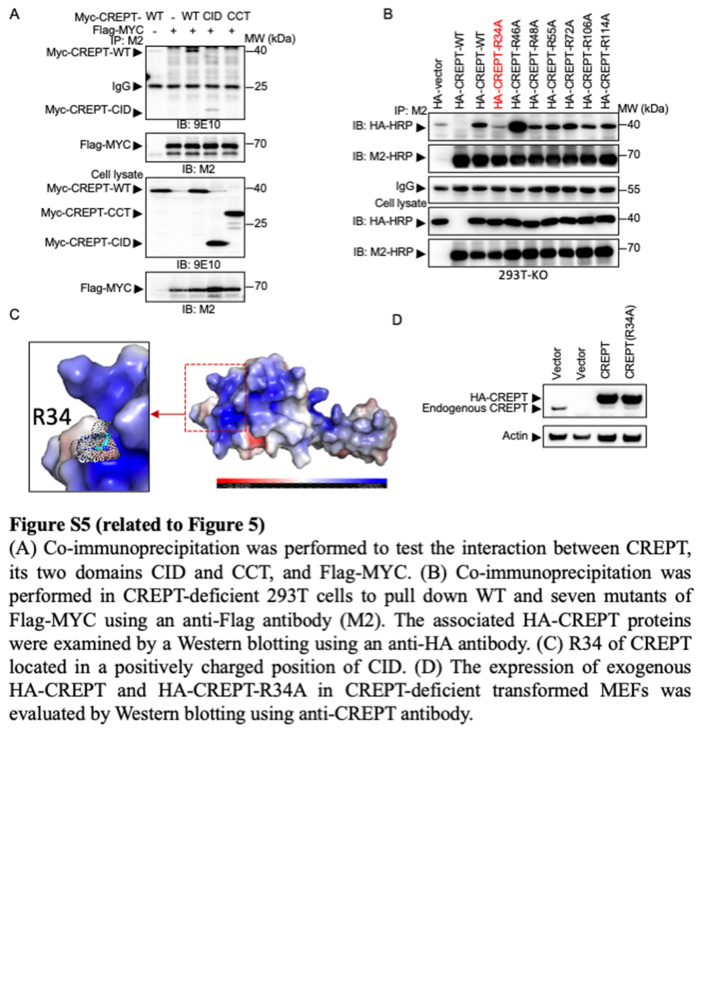


To reveal the specific residues mediating the interaction with MYC, we generated mutants of CREPT with the CID or CCT domain. An immunoprecipitation experiment showed that CID, but not the CCT domain, was responsible for the interaction of CREPT with MYC (Fig. S5*A*). Considering that the PEST domain of MYC carries negative charges and is instrumental in CREPT binding, we speculated that the positively charged arginine residues in CREPT might be docked. This was tested by expressing several CREPT mutants in a CREPT-deficient 293T cell line (293T-KO). Indeed, an immunoprecipitation assay indicated that the CREPT mutant (HA-CREPT-R34A) displayed markedly diminished binding ability to Flag-tagged MYC, while WT CREPT maintained a strong interaction (Fig. 5*A*). Notably, other arginine mutants (R48, R55, R72, R106, and R114) appeared to have a weak impact on the interaction (Figs. 5*A* and S5*B*, last five lanes). These results were confirmed by an immunoprecipitation experiment using an M2 antibody (Fig. S5*B*). Surprisingly, the R46 mutant consistently enhanced this interaction (Figs. 5*A* and S5*B*, lane 5). Nevertheless, these experiments collectively suggest that R34 is essential for the binding of CREPT to MYC. An electrostatic potential map of CREPT, as visualized with PyMOL, showed that R34 was located within a prominent positively charged area in the CID domain (Fig. S5*C*), further corroborating our findings that passively charged R34 is docked to the PEST domain. Based on this finding and previous studies on the interaction of CREPT with the CTD domain of RNAPII *via* D65, R106, and R114 [17], we built a model in which CREPT mediates the interaction of MYC and RNAPII *via* the CID domain, R34 is responsible for the PEST domain interaction, and D65, R106, and R114 are responsible for the association with the CTD domain of RNAPII (Fig. 5*B*).

To address the functional significance of CREPT-MYC interaction, we introduced the CREPT and CREPT-R34 A mutant into CREPT-deficient and MYC-transformed MEFs (Fig. S5*D*). Colony formation assays showed that CREPT deficiency (CREPT-KO) markedly reduced colony numbers in MYC-transformed MEFs (Fig. 5*C*, compare top two panels). Furthermore, while overexpression of CREPT restored colony formation, overexpression of CREPT-R34A had no effect on colony formation (Fig. 5*C*, bottom two panels). Statistical analysis demonstrated a significant difference in colony numbers restored by CREPT but not CREPT-R34A under CREPT-deficient conditions (Fig. 5*D*). Additionally, cell proliferation was not rescued by CREPT-R34A overexpression in CREPT-deficient or MYC-transformed MEFs (Fig. 5*E*). Taken together, these results suggest that the interaction between CREPT and MYC mediated by R34 is important for colony formation and proliferation of MYC-transformed MEFs.

### Deletion of CREPT reduces the recruitment of RNAPII to the MYC binding element

**Figure 6:**
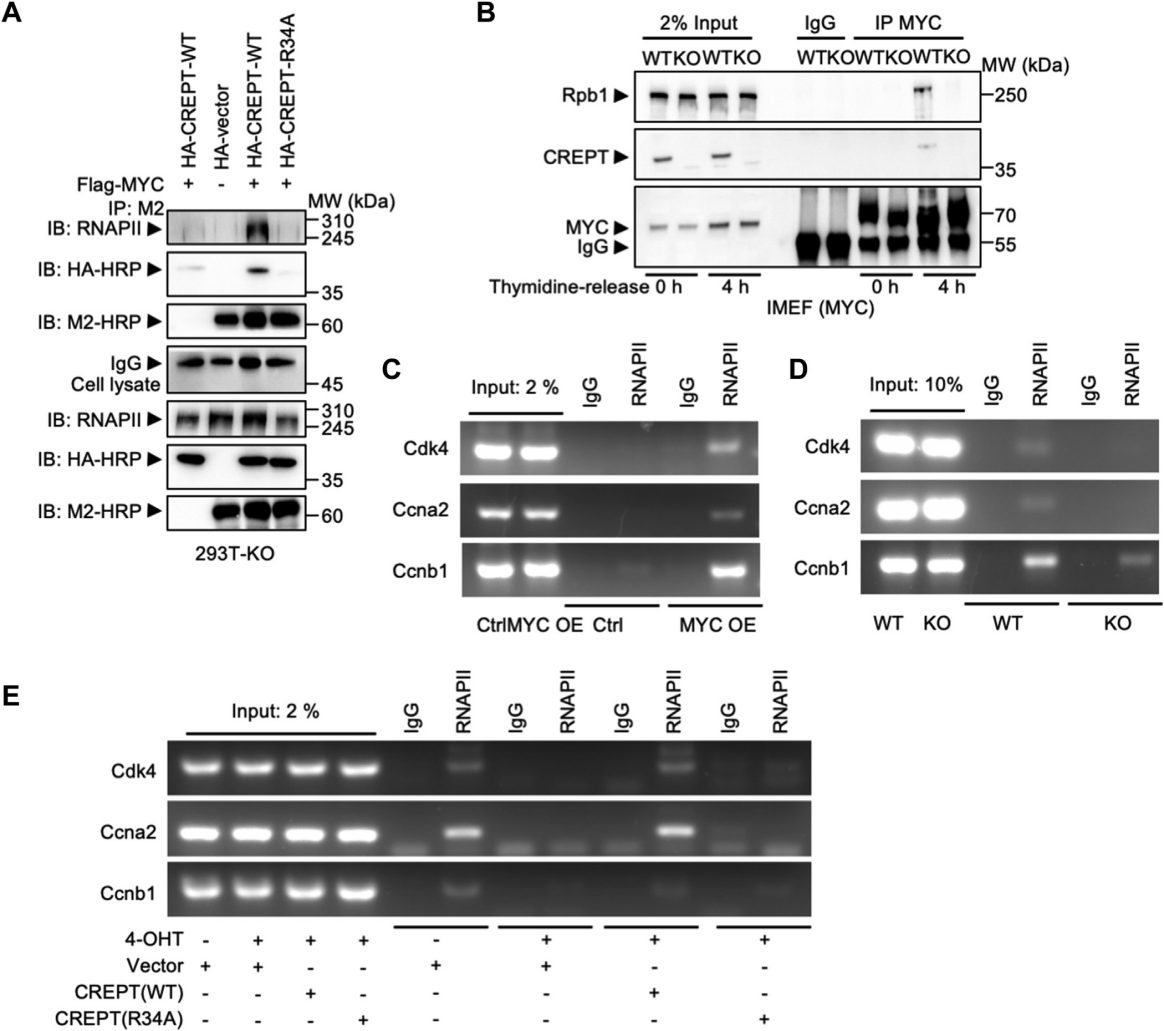
**Deletion of CREPT reduces the recruitment of RNAPII to MYC.***A*, a co-immunoprecipitation assay to evaluate the interaction between Flag-MYC and Rpb1 in the presence of HA-CREPT WT or R34A in CREPT-KO 293T cells. *B*, the endogenous interaction between MYC and Rpb1 was reduced in CREPT-KO DLD-1 cells compared to WT cells. *C*, a ChIP assay demonstrated that MYC over-expression induced RNAPII occupancy at the promoters of *Cdk4*, *Ccna2*, and *Ccnb1*. *D*, a ChIP assay showed that CREPT KO reduced RNAPII occupancy in at the promoters of *Cdk4*, *Ccna2*, and *Ccnb1* in MYC-transformed MEFs compared to CREPT WT control cells. *E*, A ChIP assay revealed that CREPT(R34A) failed to restore the RNAPII occupancy at the promoters of *Cdk4*, *Ccna2*, and *Ccnb1*.

**Fig. S6:**
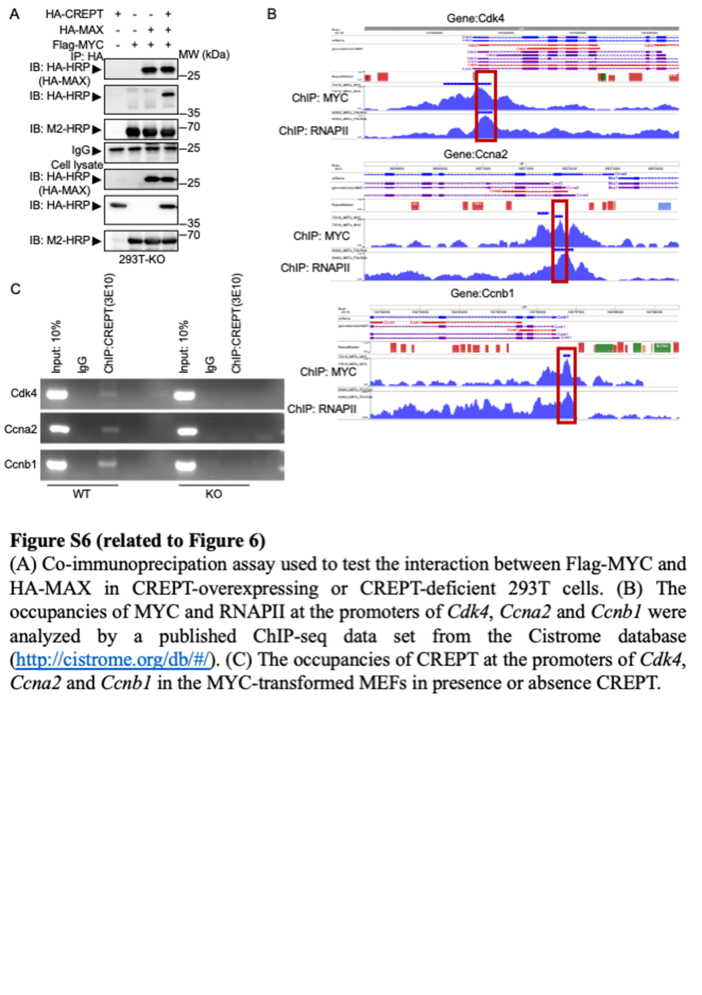


To investigate the regulatory role of the CREPT-MYC interaction in gene transcription, we examined the MYC-MAX heterodimer required to activate gene transcription (23), and its association with RNAPII. An immunoprecipitation assay revealed that overexpression of HA-tagged CREPT did not affect MYC-MAX interaction (Fig. S6*A*). Subsequently, we examined whether CREPT affected the association of RNAPII with MYC, which was docked in the aforementioned analyses (see Fig. 5*B*). Notably, the results showed that overexpression of CREPT significantly enhanced MYC-RNAPII interaction, but CREPT-R34A, which loses the ability to bind MYC, had no effect (Fig. 6*A*). Furthermore, we showed that the interaction between MYC and RNAPII was almost abolished upon the deletion of CREPT in MEFs (Fig. 6*B*), indicating that CREPT is essential for complex formation at transcriptional initiation. Given that CREPT-R34A failed to facilitate MYC-RNAPII complex formation, we concluded that CREPT directly mediates the interaction between MYC and RNAPII.

To address whether the CREPT-mediated MYC-RNAPII interaction occurs in the promoters of MYC target genes, we analyzed a published ChIP-seq dataset from the Cistrome database (http://cistrome.org/db/#/). The results showed that the occupancy of RNAPII and MYC was mapped to the MYC target genes Cdk4, Ccna2, and Ccnb1 (Fig. S6*B*). To verify the presence of CREPT in the RNAPII and MYC co-occupied regions, a ChIP assay was conducted. CREPT occupied these regions (Fig. S6*C*). We found that MYC overexpression dramatically increased the enrichment of RNAPII at the promoter regions of these genes (Fig. 6*C*). Reciprocally, the deletion of CREPT significantly diminished RNAPII occupancy (Fig. 6*D*). To determine whether CREPT-R34A affects RNAPII occupancy at the promoters, we reintroduced either wild-type CREPT or CREPT-R34A into CREPT-deleted cells driven by 4-OHT. Intriguingly, we observed that the reintroduction of wild-type CREPT, but not CREPT-R34A, restored the occupation of RNAPII at these promoters (Fig. 6*E*). Taken together, these results suggest that CREPT promotes the efficient recruitment of RNAPII to MYC-binding promoters, implying a regulation of transcription initiation of MYC-targeted genes.

### The interaction of CREPT and MYC exacerbates colorectal cancer progression

**Figure 7:**
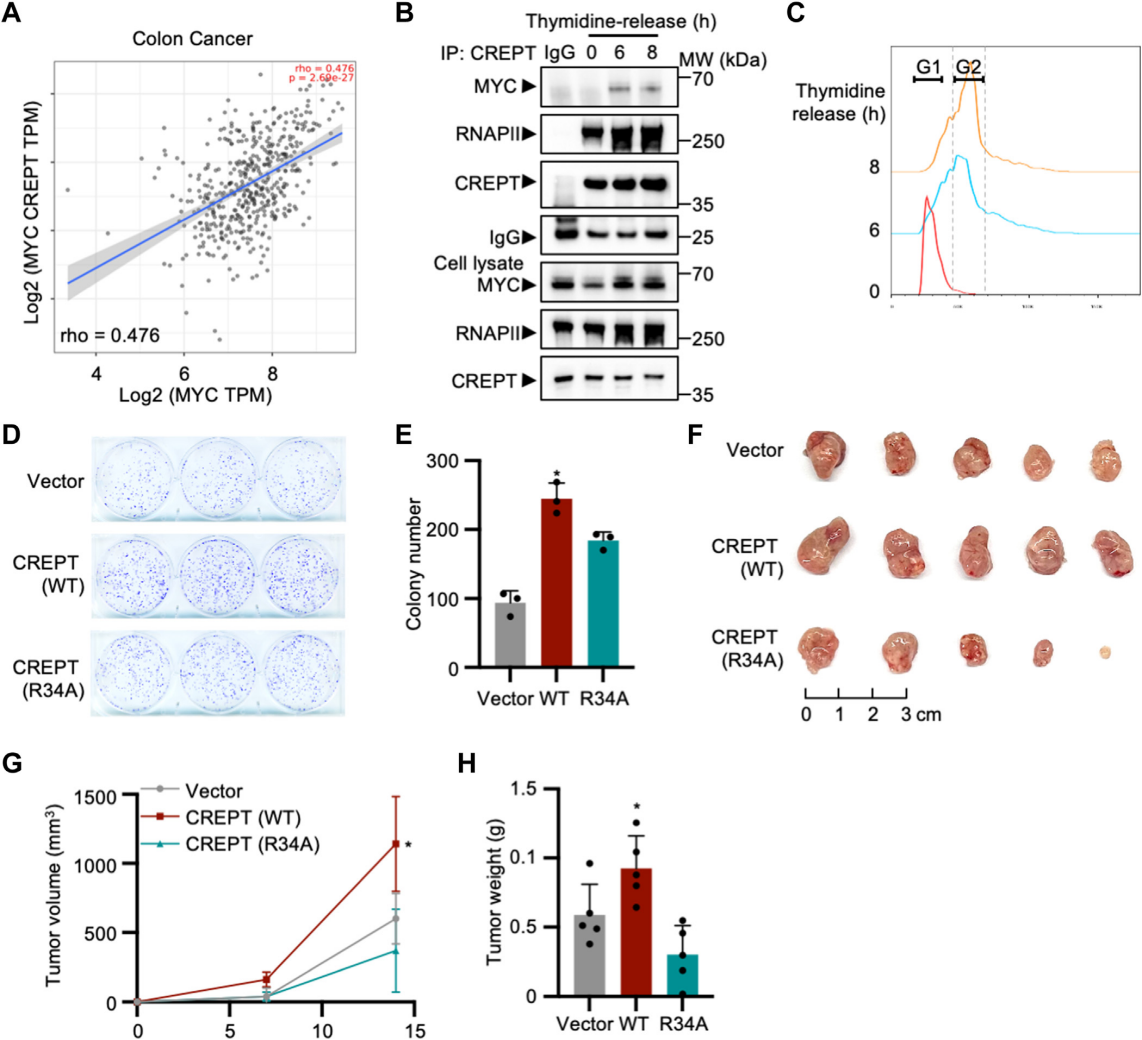
**CREPT exacerbates colorectal cancer progression with high expression of MYC.***A*, Spearman correlation coefficient (rho) between CREPT and MYC in patients with colon cancers using TIMER2.0 database (http://timer.cistrome.org/). *B*, a co-immunoprecipitation assay using anti-CREPT antibody was used to evaluate the *in vivo* interaction between endogenous CREPT and MYC in HCT116 cells released from double thymidine treatment. *C*, HCT116 were released from DTB treatment for indicated time points. The cell cycle was determined using PI staining and analyzed by flow cytometry and FlowJo. *D* and *E*, colony formation of HCT116 cells over-expressing CREPT or CREPT(R34A), along with a quantification analysis. Each column of the bar graph represents the mean ± S.D. of three biological replicates (n = 3). ∗*p* < 0.05; *p* values were obtained by unpaired, two-sided t-tests. *F*, tumor formation of HCT116 cells over-expressing CREPT or CREPT(R34A) in NU/NU nude mice using xenograft model. *G* and *H*, tumor volumes and weight were assessed in NU/NU nude mice subcutaneously injected with HCT116 cells over-expressing CREPT or CREPT(R34A). Each groups contain five mice, and the quantitative analysis was shown as the mean ± SD of five biological replicates (n = 5). ∗*p* < 0.05; *p* values were obtained by unpaired, two-sided t-tests.

To address the role of CREPT-MYC interaction on tumorigenesis, we assessed the co-expression pattern of CREPT and MYC in various human tumors from The Cancer Genome Atlas (TCGA) utilizing TIMER2.0 database (http://timer.cistrome.org/) (24). The results demonstrated a strong positive correlation (rho > 0.4) in the expression levels of CREPT and MYC in six cancers, including UVM, UCEC, COAD, SKCM, ACC, and READ, and a moderate correlation (rho > 0.2) in 18 cancers, including PRAD, STAD, GBM, and LUAD, although a weak correlation (rho < 0.2) was observed in other cancers (Fig. S7*A*). In particular, we found that the expression of CREPT and MYC correlated with colon cancer, with rho = 0.476 (Fig. 7*A*). These findings echo our aforementioned results that CREPT was upregulated in MYC-transformed MEFs (Fig. 1*C*) and downregulated upon MYC deletion (Fig. S3*A*). Taken together, we concluded that CREPT and MYC are both highly expressed in different cancers, implying their possible interaction during tumorigenesis.

To investigate the interaction between CREPT and MYC during different cell cycle stages, we performed an immunoprecipitation experiment in synchronized colon cancer cell lines. We observed that the interaction between CREPT and MYC varied in synchronized HCT116 and DLD-1 cells, with a peak during the G2/M phase (Fig. 7, *B* and *C*, 6 h in HCT116; and Fig. S7*B*, 8 h in DLD-1). These results suggest that the interaction between CREPT and MYC most likely occurs in the S and G2/M phases.

To address whether the interaction between CREPT and MYC plays a role in tumorigenesis, we examined colony formation and tumor growth using xenograft models. The results showed that CREPT overexpression markedly promoted, but CREPT-R34A failed to induce colony formation in HCT116 and SW620 cells (Figs. 7, *D* and *E*; Fig. S7, *C*–*E*). A tumorigenicity assay further revealed that overexpression of CREPT significantly enhanced tumor growth, as evaluated by the tumor volume and weight of HCT116 cells (Fig. 7, *F*–*H*, see WT vs. vector). However, we observed that overexpression of CREPT-R34A lost the ability to promote tumor growth, seemingly reducing tumor size and weight (Fig. 7, *F*–*H*, WT vs. R43A). Collectively, these results suggest that the interaction between CREPT and MYC is critical for the tumorigenesis of colon cancer cells.

## Discussion

**Figure 8:**
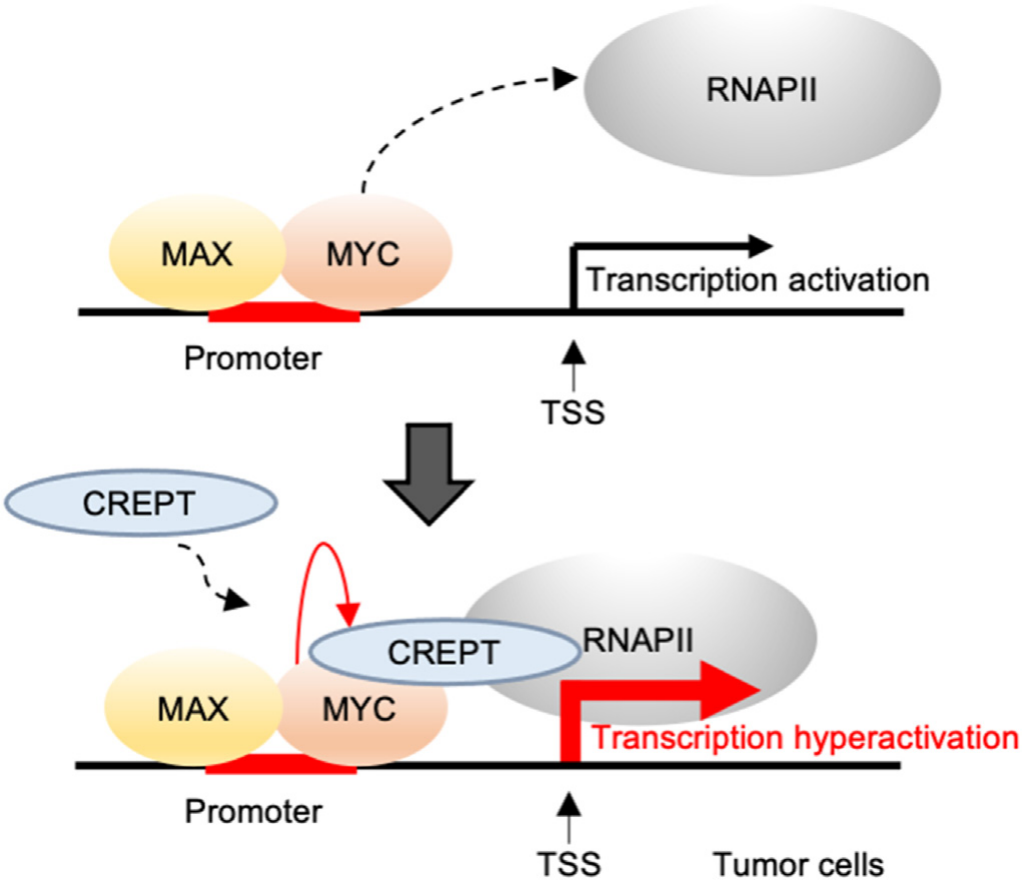
**Hyperactivation of MYC targeted gene transcription in CREPT highly expressed tumor cells.** MYC and MAX form a complex that binds to the promoters of downstream gene (*top* panel). When CREPT is highly expressed, it interacts with the MYC-MAX complex and then bridges their association with RNAPII. In such a way, CREPT assists MYC for hyperactivation of gene transcription in cancer cells.

The *MYC* proto-oncogene is associated with the development of most cancers in humans. In particular, pathological activation of MYC results in uncontrolled tumor growth (25), whereas strategies that inhibit MYC, either directly or indirectly, have been shown to cause a significant reduction in tumor size (4, 26, 27). Accumulating evidence suggests that MYC regulates a variety of oncogenic genes that trigger cancer cell proliferation, evasion, metabolic alteration, and even immune cell dysfunction in the tumor microenvironment. At the molecular level, MYC regulates gene expression *via* a complex with Max, which forms a heterodimer that binds to DNA. However, the intricate mechanisms by which the MYC-MAX complex facilitates RNAPII recruitment to gene promoters remain to be fully elucidated. In this study, we found that CREPT, an oncoprotein ubiquitously overexpressed in various cancers, serves as a critical mediator of MYC in initiating gene transcription. We propose that CREPT mediates the association between MYC-MAX and RNAPII. We provide evidence underscoring the pivotal role of the CREPT-mediated association of MYC-MAX with RNAPII in cancer development. Interestingly, we observed that CREPT was a co-factor for MYC during the transformation of MEFs. We concluded that CREPT, by interacting with both MYC and RNAPII, bridged the MYC-MAX transcriptional complex to RNAPII for gene transcription initiation (Fig. 8). Given the ubiquitous high expression of MYC and CREPT in various tumors, we speculate that targeting this complex could be a new strategy for anti-cancer therapy.

Previous studies have shown that CREPT enhances the G1/S and G2/M transitions of various human cancer cells by promoting CCND1 (10) and CCNB1 (20) expression during tumor growth (20, 23, 28). In this study, we found that deletion of CREPT resulted in G1/S and G2/M arrest and affected S phase progression in MYC-transformed MEFs. This result was consistent with our previous observations. We observed that the interaction between CREPT and MYC mainly occurred in the G2/M phase. It appeared that the interaction of CRPET and MYC regulated MYC target genes, including Ccna2 and Ccnb1 but not Ccnd1. Indeed, the MYC target genes Ccna2 and Ccnb1 were highly expressed in the S and G2/M phases, whereas Ccnd1 was mainly expressed in the G1 phase. Furthermore, we demonstrated that the CREPT target genes encoded a set of proteins that regulate the cell cycle, focal adhesion, metabolism, and Hippo pathway (Fig. S3*B*). This is consistent with other observations that MYC regulates fatty acid metabolism, the cell cycle, and focal adhesion *via* modulating genes involved in steroid biosynthesis, cyclin-dependent kinase, integrins, and focal adhesion kinase (29, 30, 31, 32). These results imply that oncogenic proteins that transform a normal cell by MYC mainly function in the G2/M phase. This echoes our previous study showing that CREPT is phosphorylated by Aurora B and regulates the malignant features of tumors (20). Our study provides evidence that CREPT and MYC are critical regulators of the malignant transformation of tumor cells. Whether Aurora B regulates the interaction between CREPT and MYC will be of interest for future studies.

We demonstrated that CREPT binds to the PEST domain of MYC. PEST sequences are enriched with proline (P), glutamic acid (E), serine (S), threonine (T), and aspartic acid (D), which are often found in short-lived proteins (33). The PEST domain extends to residues 201 and 268 of MYC. The role of the PEST domain has not been well studied, but it has been recognized as a regulator of rapid proteolysis (34). This domain remains of a low complexity and lacks a stable tertiary structure (33, 35). Interestingly, this is very similar to the CTD of RNAPII, which is a long unstructured domain containing tandem repetitions of the heptapeptide with low complexity (36). Therefore, we speculate that CREPT interacts with these two flexible domains to stabilize MYC and RNAPII complexes. To the best of our knowledge, no previous study has reported a direct interaction between MYC and RNAPII. Our study revealed an important mechanism by which MYC is associated with RNAPII by CREPT during tumorigenesis. We defined that CREPT interacted with MYC *via* R34, which is the distance to residues D65, R106, and R114 responsible for the interaction with the CTD domain in RNAPII (17) (see Fig. 5*B*). In conclusion, we propose that CREPT bridges the association between MYC and RNAPII *via* flexible PEST and CTD domains.

However, unlike the binding between CREPT and RNAPII, our observations indicated that the interaction between CREPT and MYC is more likely to occur during the late S and G2/M phases of the cell cycle in both transformed MEFs and human tumor cells. However, the precise underlying mechanisms are not completely understood. The binding may likely be influenced by a combination of factors, including the conformational changes in MYC or CREPT during the cell cycle. Notably, MYC regulation during the cell cycle is complex and involves multiple pathways and proteins that interact with it at various stages (5, 37). During the cell cycle, MYC undergoes a variety of post-translational modifications, including phosphorylation, acetylation, and ubiquitination, which may affect its structure and function. These modifications are carried out by various kinases and phosphatases that are differentially expressed and active during different stages of the cell cycle (38, 39). It is possible that the binding of CREPT to MYC is regulated by the specific modifications that MYC undergoes during the S and G2/M phases.

We demonstrated that CREPT and MYC are co-expressed in several cancers. How these two genes were coordinately regulated during the tumorigenesis remains unclear. In a previous study, we observed that CREPT regulates MYC expression (15). However, in this study, we observed that the deletion of CREPT failed to affect MYC expression in MYC-transformed MEFs (see Fig. S2*A*). We speculate that this might be because the exogenous expression of MYC is driven by the CMV promoter, which has a strong ability to drive gene expression, and the endogenous expression of MYC is low in MEFs. During the development of tumors, CREPT may regulate endogenous MYC expression, as previously observed (15). In contrast, MYC may also regulate CREPT expression. Indeed, in a recent study, we observed that the promoter of CREPT contained an E-Box, which is an MYC-binding site (manuscript submitted). Therefore, we propose that the coordination of CREPT and MYC expression may be critical for tumorigenesis.

In conclusion, we reported that the interaction between CREPT and MYC initiates the expression of oncogenic genes by activating RNAPII. This process is critical for the promotion of tumorigenesis in colorectal cancer and other cancers. This study sheds light on the mechanisms underlying MYC regulation of cancer development and may have implications for developing targeted therapies against cancer.

## Method

### Mouse embryonic fibroblasts

MEFs were obtained from CreERT2^+/−^;CREPT ^flox/flox^, EIIαCre^+^, or EIIαCre^+^; TgCREPT mice, which were all maintained in a specific pathogen-free facility. The use of these mice was approved by the AAALAC (Association for Assessment and Accreditation of Laboratory Animal Care International) and the IACUC (Institutional Animal Care and Use Committee) of Tsinghua University. MEFs were derived from multiple embryos, and all mice used in the study had a C57BL/6 genetic background. To induce the deletion of CREPT in MEFs, 1 μM 4-Hydroxytamoxifen (4-OHT) was used for 24 h.

### Plasmids

pcDH-HA-MYC was used to transform the MEFs. Human MYC coding sequences were generated from cDNA of HEK 293T (293T) cells by polymerase chain reaction (PCR) and transferred to pcDH-based HA-epitope or pcDNA3.1-based Flag-epitope vectors using a ClonExpress II one Step Cloning Kit (C112–02, Vazyme). HA-MAX was constructed by inserting MAX PCR-amplified sequences into the pcDH-HA vector. GST/HA-CREPT, Myc-CREPT-RPR, and Myc-CREPT-CCT were previously constructed in our lab (10, 40). All mutations were introduced using a Muta-Direct Kit following the manufacturer’s protocols (SDM-15, Saibaisheng).

### Cell culture and transfection

MEFs and 293T cells were cultured in Dulbecco’s modified Eagle’s medium (DMEM), while HCT116, SW620, and DLD-1 cells were maintained in Roswell Park Memorial Institute (RPMI) 1640 Medium and SW620 cells were cultured in Leibovitz's L-15 Medium. All media were supplemented with 10% fetal bovine serum (Gibco), 100 U/ml of penicillin, and 100 mg/ml of streptomycin (Gibco). Cells were incubated at 37 °C with 5% CO_2_ for 293T and DLD-1 cells, and without CO_2_ for SW620 cells. Plasmids were transfected into cells using Vigofect (#T001, Vigorous Inc). Deletion of CREPT in DLD-1 cells was achieved as previously reported (20). Cell lines were verified by DNA fingerprinting (short tandem repeat/STR examination). All cell lines were tested for the presence of *mycoplasma* and found to be negative. MYC depletion was achieved by using two siRNAs, siRNA-1: sense, 5′-CTTAAGTGGGTTCTCACTCA-3′; anti-sense, 5′-UCCAAGACGUUGUGUGUUCTT-3′ and siRNA-2: sense, 5′- TCCATATGTGTTCGGCTCTC-3′; anti-sense, 5′-AACUGUUCUCGUCGUUUCCTT-3′.

### Stable MYC-transformed MEF cell line

To generate lentivirus for the expression of HA-MYC, psPAX, and pMD2.G packaging plasmids with pcDH-HA-MYC were transiently transfected into 293T cells. Lentiviral supernatants were collected 48 h after transfection and filtered through a 0.45 μm filter. For infection, the parent cells were incubated with the virus supernatant for 48 h and split into selection media containing 2 μg/ml puromycin (P8230, Solarbio). Selection media were replaced every 2 to 3 days until the selection was completed.

### Cell cycle synchronization

Cells were synchronized at the G1/S phase using a double thymidine block (DTB) or DMEM supplemented with 0.5% FBS. To perform DTB, the cells were treated with thymidine (2 mM, T1895, Sigma-Aldrich) for 18 h, released into fresh medium for 9 h, washed three times with PBS, and then treated again with thymidine for 16 h.

### Transformation, cell proliferation, and apoptosis assays

Transformation ability was assessed through either anchorage-dependent or anchorage-independent colony formation assays. For anchorage-dependent colony formation, cells were seeded in 6-well plates at a density of 500 to 1000 cells per well and cultured for 1 to 3 weeks before staining with 0.01% crystal violet in 40% methanol diluted in PBS. For anchorage-independent colony formation, cells were suspended in a complete medium supplemented with 0.3% low-melting agarose and seeded in 6-well plates at 10,000 cells per well on solidified 0.7% agarose. The top layer of the soft agar was refreshed with fresh culture medium twice a week, and the colonies were stained with 0.005% crystal violet in 40% methanol diluted in PBS. The cell proliferation was determined by counting the number of cells. Cells were plated in 6-well plates at 1000 cells per well and counted using a hemocytometer. Cell viability was assessed using the Cell Counting Kit-8 (CCK-8, CK04, DOJINDO Laboratories), and cell apoptosis was tested using a FITC Annexin V Apoptosis Detection Kit with PI (#640914, BioLegend) according to the manufacturer's instructions.

### Flow cytometric analyses

The cell cycle was determined by propidium iodide (PI) staining or EdU staining. The cells were fixed in cold 70% ethanol. For 1.5 ml cell suspension, 50 μl of RNase (100 μg/ml, RT405–12, TIANGEN), and 200 μl PI (50 μg/ml, CD057, MACGENE) were added. For EdU staining, cells were labeled with 10 μM EdU for 2 h, fixed, permeabilized, and detected with a fluorescent azide probe using a Click-iT Plus EdU Flow Cytometry Assay Kits (C10634, Invitrogen). The stained cells were analyzed by flow cytometry.

### Immunofluorescence staining

MEFs were seeded onto coverslips and fixed with 4% paraformaldehyde for 20 min, followed by permeabilization with 0.5% Triton X-100 for 10 min. To block non-specific binding, cells were incubated with 10% FBS for 1 h and then incubated overnight incubation with primary antibodies anti-CREPT or anti-MYC (#9402, CST) at 4 °C. The cells were subsequently incubated with FITC-conjugated secondary antibodies (green) or TRITC-conjugated antibodies (Jackson Research Laboratories) for 1 h and the nuclei were stained with DAPI (D8417, Sigma-Aldrich). Images of stained cells were acquired using an FV3000 laser scanning microscope (Olympus).

### Quantitative RT-PCR and RNA sequencing

RNA was extracted from the cells using TRIzol reagent (Invitrogen) according to the manufacturer’s protocol. The cDNA was prepared using from 2 μg of total RNA using a kit (TIANGEN). Real-time PCR was performed using SuperReal PreMix Plus (TIANGEN) on a LightCycler480II Real-time PCR instrument (Roche). Data were analyzed using the 2^–ΔΔCt^ method and normalized to β-actin levels. For RNA-seq analysis, 2 μg RNA was submitted to BioMarker for library construction, sequencing, and data processing. Primers used for qRT-PCR are listed in Table S1.

### Immunoprecipitation and western blotting

Cells were transfected with the indicated plasmids for 24 h to induce exogenous protein expression in 293T cells before being lysed. For endogenous interaction assays, cells were directly lysed in radioimmunoprecipitation assay (RIPA) lysis buffer (50 mM Tris-Cl, 150 mM NaCl, 1% Nonidet P-40, 0.5% sodium deoxycholate, and 1% SDS, pH 8.0) supplemented with freshly added protease inhibitors. Protein A/G-agarose beads were used for immunoprecipitation with specific antibodies. Western blotting was used to detect the precipitated proteins with specific antibodies and 5% of cell lysates were used. For western blotting, the samples were loaded onto a 10% SDS-PAGE gel and then transferred onto nitrocellulose blotting membranes. The following antibodies were used: anti-CREPT (3E10, produced in this lab), anti-M2 (F1804, Sigma-Aldrich), anti-HA (sc-7392, Santa, USA), and anti-MYC (#9402, CST), anti-β-actin (#3554, Sigma-Aldrich), anti-HA-HRP (#14031, CST), anti-H3S10p (ab8898, Abcam), anti-H3 (#9715, Cell Signaling Technology), anti-M2-HPR (M2, ab49763, Abcam, USA), anti-M2(F9291, Sigma-Aldrich), anti-Max (4739s, Cell Signaling Technology), anti-RNAPII (#2629, CST) and anti-9E10 (Myc tag, produced in this lab). Immunoreactive bands were visualized using a SAGECREATION system after developing them with an ECL kit (Thermo Fisher Scientific). The specificity of each antibody was validated using overexpression of the specific protein or selected based on their use in previous studies.

### *In vitro* protein binding assay

GST-CREPT proteins were purified from *E. coli* (40). For eukaryotic protein purification, GST-CREPT or Flag-MYC plasmids were transfected into 293T cells for 48 h, and the cells were lysed using RIPA buffer containing protease inhibitors. GST-tagged Sepharose beads (Amersham Pharmacia Biotech) were used to pull down GST-CREPT and were eluted using elution buffer (50 mM Tris-HCl, 10 mM reduced glutathione, pH 8.0). Flag-MYC was purified using Anti-Flag M2 Affinity Gel (A2220, Sigma-Aldrich) and eluted by adding 150 ng/μl of 3X FLAG peptide (F3290, Sigma-Aldrich). For *in vitro* protein binding assay, 4 μg of purified GST-CREPT proteins and 4 or 8 μg of purified Flag-MYC were incubated at 4 °C overnight. GST-tagged Sepharose beads were used to pull down the complex and anti-M2 was used to detect the binding of Flag-MYC proteins.

### Xenograft tumor formation assay

NU/NU nude mice aged 6 to 8 weeks were divided into three groups (n = 5 for each group), which were housed in the Tsinghua University Association for Assessment and Accreditation of Laboratory Animal Care International (AAALAC)-accredited facility with a 12-h light-dark cycle and temperature control between 22 °C and 26 °C. The mice had *ad libitum* access to sterile food and water. All animal protocols were approved by the IACUC (Institutional Animal Care and Use Committee) of Tsinghua University, China. Each mouse was subcutaneously injected into the right axillary region with 2.5 × 10^6^ HCT116 cells expressing three different constructs: GFP-HA-vector (control), GFP-HA-CREPT, and GFP-HA-R34A. Tumors were measured each week and tumor volumes (a^2^ b/2) were calculated. After 2 weeks, all mice were sacrificed, and the tumors were excised and weighed using a precision analytical balance.

### Statistical analysis

All experiments described in this study were independently performed three times. Two replicates were used for the RNA-seq experiments. The results of RNA sequencing were analyzed using Deseq2 for differential gene expression analysis and ClusterProfiler packages for KEGG and Gene Set Enrichment Analysis (GSEA) in R (version 4.2.2). PyMOL (version 2.5.2) was used to predict the surface electrostatic potential of the CID domain of CREPT and the model of protein interaction. The Spearman correlation (rho) between CREPT and MYC in various human tumors from The Cancer Genome Atlas (TCGA) was analyzed utilizing TIMER2.0 database (http://timer.cistrome.org/) (24). All data are expressed as mean ± standard deviation (SD). Statistical analyses were performed using Student's *t* test to determine the significant differences between the two groups. Unpaired, two-sided t-tests were used for normally distributed data. A value of *p* less than 0.05 was considered statistical significance. ∗*p* < 0.05; ∗∗*p* < 0.01; ∗∗∗*p* < 0.001; ∗∗∗∗*p* < 0.001.

## Data availability

Data supporting the findings of this study are available from the corresponding author upon reasonable request.

## Supporting information

This article contains supporting information.

## Conflict of interest

The authors declare that they have no conflicts of interest with the contents of this article.

## Figures and Tables

**Fig. S7:**