# Supplementary material for: An oncoprotein CREPT functions as a co-factor in MYC-driven transformation and tumor growth
Source: J Biol Chem. 2024 Nov 29;301(1):108030. doi: 10.1016/j.jbc.2024.108030 (PMC11730240; doi:10.1016/j.jbc.2024.108030)
Supplement: Table S1 [file mmc1.docx]

Supplementary Table 1 The primers for quantitative RT-PCR

| Primer for mouse cell | 5'-3' |
| --- | --- |
| CDK4-Forward | TGGAAACTCTGAAGCCGACC |
| CDK4-Reverse | TTCTCACTCTGCGTCGCTTT |
| CCNE1-Forward | TGGTTATCCGGGAGATGGGA |
| CCNE1-Reverse | CTTCTTACTGCTGGGTGGGG |
| CCNB1-Forward | TGCATTTTGCTCCTTCTCAA |
| CCNB1-Reverse | CAGGAAGCAGGGAGTCTTCA |
| CCNA2-Forward | TGCAAACTGTAAGGTTGAAAGC |
| CCNA2-Reverse | TGTAGAGAGCCAAGTGGAAGG |
| Primer for human cell | 5'-3' |
| MYC-F | TCATAACGCGCTCTCCAAGTA |
| MYC-R | AAATCATCGCAGGCGGAACA |
| CDK4-Forward | GTGTATGGGGCCGTAGGAAC |
| CDK4-Reverse | CAGTCGCCTCAGTAAAGCCA |
| CCNE1-Forward | CCATCATGCCGAGGGAGC |
| CCNE1-Reverse | TAATCCGAGGCTTGCACGTT |
| CCNB1-Forward | GAAACGCATTCTCTGCGACC |
| CCNB1-Reverse | TGCCATGTTGATCTTCGCCT |
| CCNA2-Forward | GCACTGGTGGTCTGTGTTCT |
| CCNA2-Reverse | ATGCCAGTCTTACTCATAGCTGAC |
